# Supplementary figures and images for: Antiglycation Activity and HT-29 Cellular Uptake of Aloe-Emodin, Aloin, and Aloe arborescens Leaf Extracts
Source: Molecules. 2019 Jun 5;24(11):2128. doi: 10.3390/molecules24112128 (PMC6600357; doi:10.3390/molecules24112128)

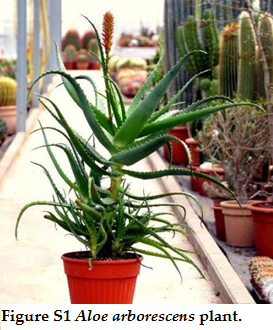

Supplement: Supplementary file 1 [file molecules-24-02128-s001.zip › Supplementary Materials Aloe R1/Figure S1 Aloe arborescens Manuscript.tif]

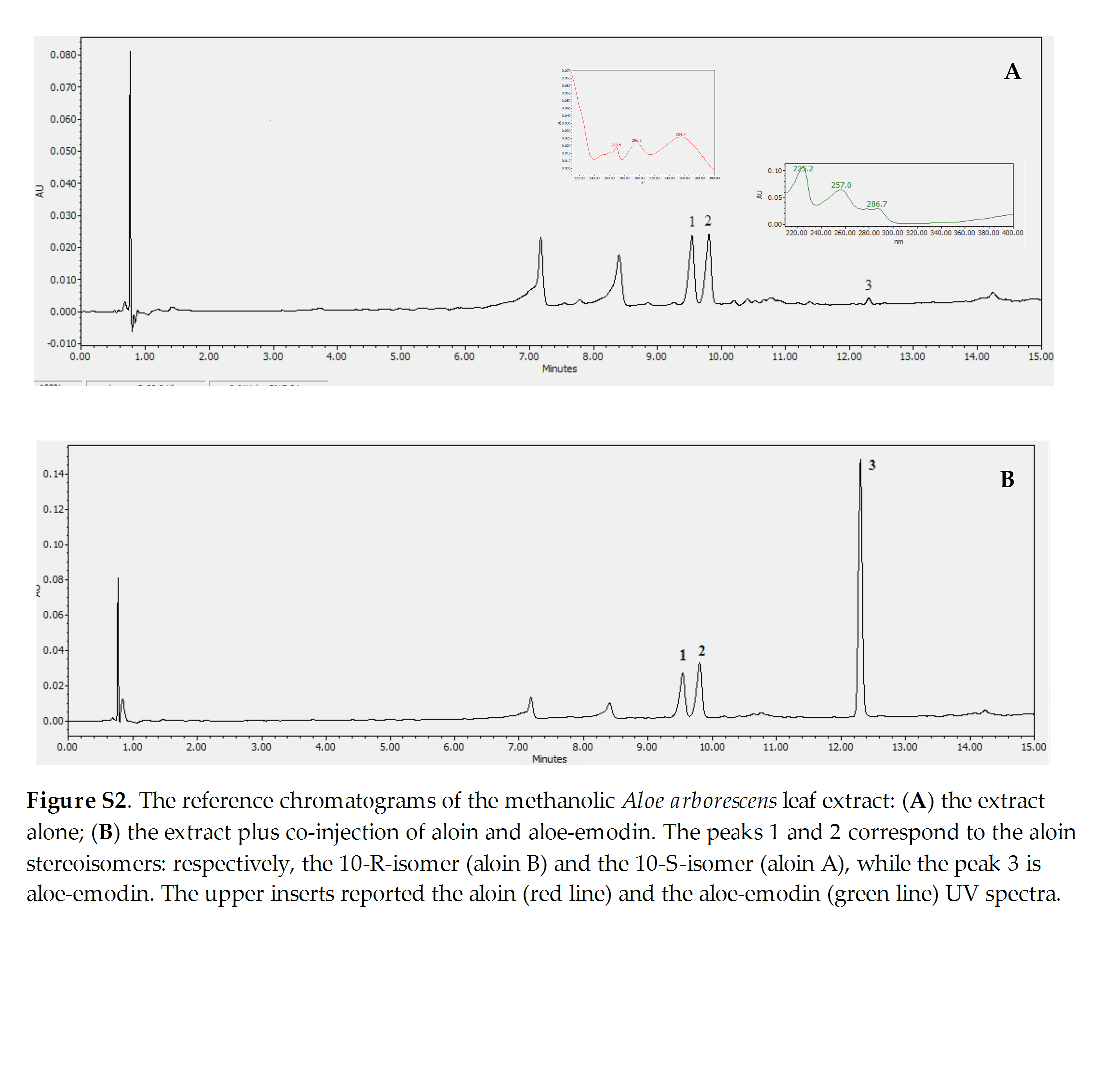

Supplement: Supplementary file 1 [file molecules-24-02128-s001.zip › Supplementary Materials Aloe R1/Figure S2 Aloe extracts chromatograms R1 .tif]

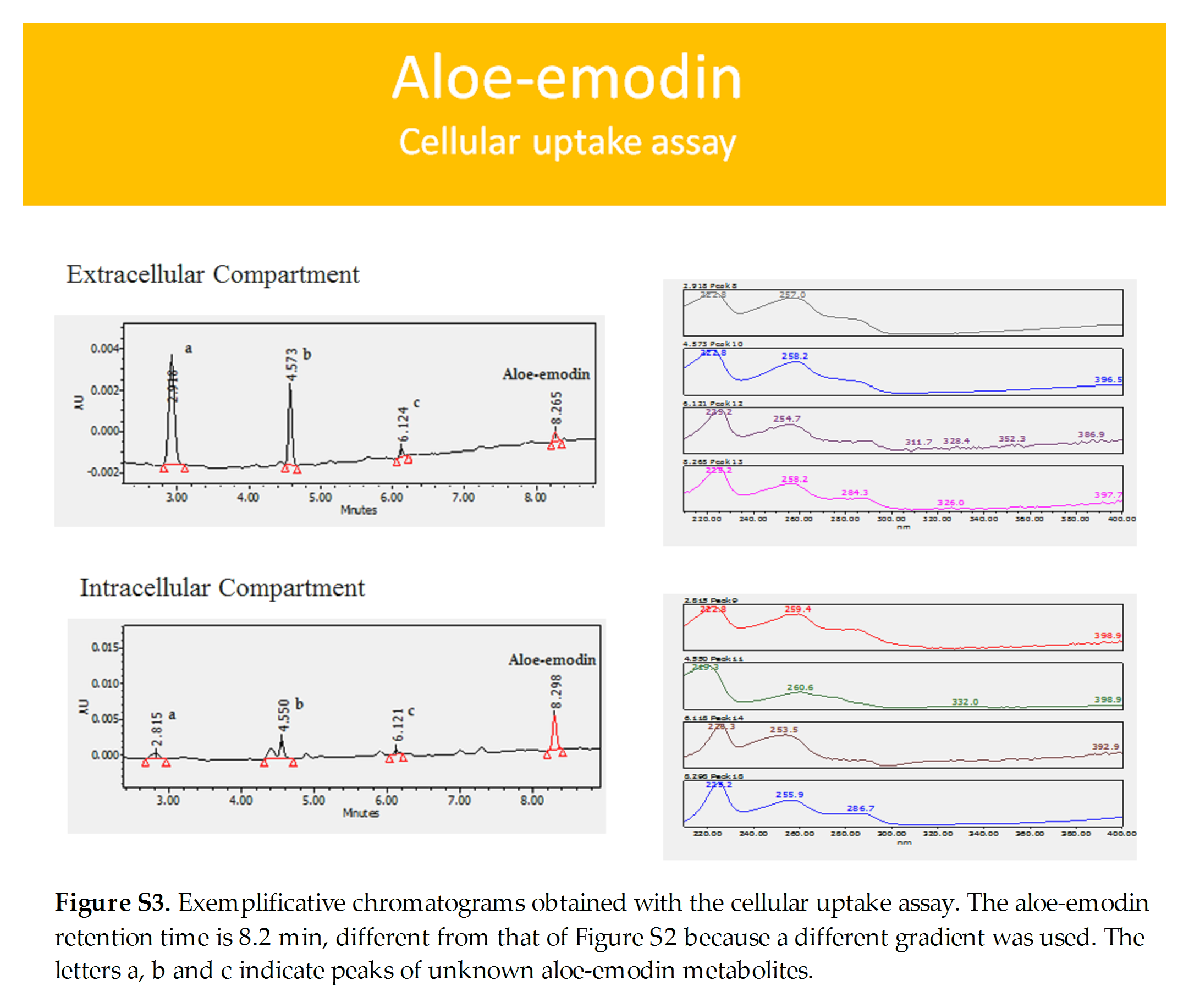

Supplement: Supplementary file 1 [file molecules-24-02128-s001.zip › Supplementary Materials Aloe R1/Figure S3 Cellular uptake Aloe R1 .tif]
